# Emission accounting and drivers in Central Asian countries

**DOI:** 10.1007/s11356-023-29608-0

**Published:** 2023-09-06

**Authors:** Congyu Zhao, Binyuan Liu, Jieyu Wang, Rui Xue, Yuli Shan, Can Cui, Xiucheng Dong, Kangyin Dong

**Affiliations:** 1 School of International Trade and Economics, University of International Business and Economics, Beijing, 100029 China; 2 School of Geography, Earth and Environmental Sciences, University of Birmingham, B15 2TT, Birmingham, UK; 3 Integrated Research on Energy, Environment and Society (IREES), Energy and Sustainability Research Institute Groningen, University of Groningen, Groningen, 9747 AG the Netherlands; 4 Guangdong Provincial Key Laboratory of Urbanization and Geo-simulation, School of Geography and Planning, Sun Yat-sen University, Guangzhou, 510006 China; 5 La Trobe Business School, La Trobe University, Melbourne, Victoria 3086 Australia; 6 Department of Earth System Science, Tsinghua University, Beijing, 100084 China

**Keywords:** CO_2_ emissions, Emission accounting, Decoupling analysis, Decomposition analysis, Central Asia

## Abstract

Emerging countries are at the frontier of climate change actions, and carbon emissions accounting provides a quantifiable measure of the environmental impact of economic activities, which allows for comparisons of emissions across different entities. However, currently there is no study covering detailed emissions inventories for emerging countries in Central Asian. This paper compiles detailed and accurate carbon emissions inventories in several Central Asian countries (i.e., Kazakhstan, Kyrgyzstan, Pakistan, Palestine, Tajikistan, and Uzbekistan) during the period 2010–2020. Using the IPCC administrative territorial approach, we for the first time compile their emissions inventories in 47 economic sectors and five energy categories. Moreover, we also investigate decoupling status based on Tapio decoupling model and examine emissions driving factors based on the index decomposition analysis method. The primary results illustrate that carbon emissions in Central Asian countries are increasing with huge differences. Decoupling results highlight that most of the sample countries still need more effort to decouple the economy and emissions except that Pakistan achieves an ideal strong decoupling state. The results of the decomposition indicate that the economy and population both raise emissions, while energy intensity and carbon intensity are negative drivers in some countries. We propose practical policy implications for decarbonization and energy transition roadmap in Central Asian countries.

**Supplementary Information:**

The online version contains supplementary material available at 10.1007/s11356-023-29608-0.

## Introduction

Low-carbon development is crucial for mitigating climate change problems (Bruckner et al. 2022; Hubacek et al. 2021; Tao et al. 2022; Tian et al. 2022; Watari et al. 2023; Wunderling et al. 2022). The latest United Nations Climate Change Conference (COP 27) in November 2022 calls for the implementation of carbon reduction measures and commitments, and also emphasizes the obstacles and demands of developing countries during their decarbonization transition process (Atwoli et al. 2022; Masood et al. 2022). To formulate feasible and effective carbon emissions reduction roadmaps, accurate carbon emissions accounting is the prerequisite (Liu et al. 2023; Sun et al. 2022; Wang et al. 2020b; Wang et al. 2019). Although carbon accounting for developed countries such as European countries and big emitters such as China have relatively complete emission data (Cui et al. 2022a, 2022b; Huang et al. 2021; Long et al. 2020; Shan et al. 2022; Xiao et al. 2021; Xiao et al. 2022), to date, carbon accounting for developing countries and emerging economies have not received much attention from academia. These include Central Asian countries where energy consumption is increasing rapidly. According to the statistics from British Petroleum (BP 2022), the last decade (e.g., 2010–2020) has witnessed fast-rising energy consumption in Kazakhstan, Pakistan, and Uzbekistan, with a strong boom from a total of 7.69 in 2010 to 9.62 EJ in 2020, indicating a growth rate of 25.15%. Rapid energy consumption inevitably leads to the growth of carbon emissions (Mohsin et al. 2022; Nguyen 2019). Therefore, it is necessary to calculate the carbon emissions of Central Asian countries. In addition, energy consumption and subsequent emissions have triggered severe climate events, manifested by the frequent occurrence of intensified weather and natural disasters in Central Asia (Lioubimtseva and Cole 2006; Liu et al. 2021b; Reyer et al. 2017; Sorg et al. 2015). For example, flash floods, landslides, and ice lake collapse caused by torrential rain in Pakistan in 2022 have ruined their peaceful life (ADB 2022). Hence, it is very urgent to develop its low-carbon development roadmap based on a more accurate calculation of carbon emissions in Central Asia.

The importance of thoroughly examining the carbon emissions of Central Asian countries is necessitated by the large variations in these countries’ ability to reduce emissions, mitigation goals, and endowment of energy resources. Specifically, some Central Asian countries have participated in the Paris Agreement and declared their emissions reduction plans in National Determined Contributions (NDCs) (Amponin and Evans 2016; Winning et al. 2019). For example, Kazakhstan promised to realize a 15% reduction in greenhouse gas emissions by 2030 unconditionally (Wang et al. 2019). By comparison, Pakistan intends to achieve a 50% reduction in emissions by 2030 conditionally with 35% depending on international financial support (Government of Pakistan 2021). However, some of them, i.e., Kyrgyzstan and Uzbekistan, failed to ratify their intended NDCs as officially recognized emission targets (Yang et al. 2020). Given the fact that these countries have already realized the urgency of emission reduction and have made specific commitments to NDCs, it is necessary to study and compare their carbon emissions. More importantly, Central Asian countries have substantial potentials for developing renewable energy, such as hydropower, solar, wind, and geothermal, with Kyrgyzstan and Tajikistan enjoying large-scale hydropower (Shadrina 2020; Zakhidov 2008); nevertheless, the deployment of these renewables is minuscule (Laldjebaev et al. 2021). Possible barriers may be backward and outdated technology and induced limited energy utilization efficiency (Gómez et al. 2015; Karimov et al. 2013). Even worse, rigid social and incumbent institutions and political risks pose additional challenges to the progress of renewables (Juaidi et al. 2016; Laldjebaev et al. 2021). Therefore, it is also critical to analyze the carbon emissions mitigation roadmap in light of their specific energy resources and energy mix.

There is a lack of comprehensive and systematic knowledge regarding the current carbon emissions situation in Central Asian countries, especially when considering studies on carbon emissions accounting in these nations. On the one hand, only a few scholars have conducted calculations specifically focused on emissions of individual countries such as Kazakhstan and Kyrgyzstan (Wang et al. 2019; Yang et al. 2020). On the other hand, some researchers have exclusively analyzed emissions within specific sectors, such as the thermal power industry in Pakistan (Yousuf et al. 2014) and the transportation industry in Kyrgyzstan (Kondev et al. 2023). This dearth of comprehensive and systematic research hinders policymakers and academics from gaining a complete understanding of the carbon emissions mitigation process in Central Asia. Thus, it is necessary to study the overall carbon emissions in Central Asian countries by sector and energy type, and make appropriate comparison analyses. Furthermore, existing research is not sufficient to support our understanding of changes in emissions and the planning of its emission reduction measures when it comes to the decoupling degree between economic growth and carbon emissions, as well as the hidden driving factors. Xiong et al. (2019) find that population scale, economic growth, and industrialization contribute to CO2 emissions increase (Li et al. 2018; Xiong et al. 2020). Wang et al. (2020a) use the logarithmic mean Divisia index (LMDI) model to identify the driving forces of territory CO2 emissions and show that economic growth and population scale accelerate CO2 emissions, while the energy intensity effect helps reduce them. Li et al. (2019) further suggest that both energy intensity effect and energy carbon structure effect inhibit CO2 emissions. Although some studies have investigated the nexus and causality among carbon emissions, energy, and economic development, they mainly adopt the econometric models without calculating a specific degree of decoupling between economic development and carbon emissions (Anser 2019; Mirza and Kanwal 2017; Rehman et al. 2021; Xiong et al. 2019). In addition, several studies have explored the driving factors behind carbon emissions, but lack comparisons among Central Asian countries and in-depth analysis, as well as an updated research period. In summary, the carbon emissions accounting in Central Asian countries and its driving factor analysis are distinct literature gaps.

Aiming at the above research gaps, our study would like to address the following research questions. First, what are the carbon emission patterns in Central Asian countries during 2010–2020? Second, what are the decoupling states between carbon emissions and GDP in these countries? Third, what are the underlying drivers of emissions growth? This paper comprehensively addresses these questions and introduces the following innovations. First, we are among the first studies to compile carbon emissions inventories in six Central Asian countries (i.e., Kazakhstan, Kyrgyzstan, Pakistan, Palestine, Tajikistan, and Uzbekistan) during the period 2010–2020. Second, for the first time, we decompose carbon emissions growth into five driving factors, namely, carbon intensity, energy consumption mix, energy intensity, economic growth, and population, which offer us insights into more detailed drivers compared with current studies.

This paper makes significant contributions by creating comprehensive and meticulous carbon emissions inventories for six Central Asian countries from 2010 to 2020. By utilizing the administrative territorial approach recommended by the Intergovernmental Panel on Climate Change (IPCC), our inventories go beyond existing studies as we provide detailed emissions data for 47 economic sectors and five energy categories. This level of granularity enables us to present a comprehensive overview of carbon emissions from each industry and energy type, a previously unexplored aspect in the region. In addition, our emission inventories offer consistency and comparability across the studied countries. This unified and normative framework for carbon emissions estimation can serve as a valuable reference for other emerging nations, facilitating their own emissions assessments over a longer time period. The six Central Asian countries examined in our study possess unique characteristics. They are landlocked nations with abundant energy resources and hold significance as emerging economies undergoing energy transition. Consequently, the findings of this paper carry crucial practical implications. We are able to propose concrete policy recommendations to facilitate low-carbon transitions and energy structure upgrades in these countries, which can extend to other emerging nations that share similar development stages, energy structures, and resource endowments as the Central Asian countries under study. Additionally, we delve into the analysis of decoupling using the Tapio decoupling model. Our findings reveal that among the Central Asian countries studied, only Pakistan has achieved the desirable state of strong decoupling, successfully disconnecting its economic growth from emissions growth. In contrast, other countries continue to face challenges in decoupling their economies from emissions. This discovery contributes to our understanding of whether the “win-win” scenario of economic growth and emissions mitigation can be attained in Central Asian countries. Furthermore, it holds great value for policymakers as they can assess the effectiveness of their green development strategies and evaluate the progress made toward achieving sustainable and low-carbon economic growth. Moreover, we conduct an examination of the driving factors of emissions using the index decomposition analysis (IDA)–LMDI method. Through the decomposition of carbon intensity, energy structure, energy intensity, economy, and population effects, we thoroughly explore the driving forces behind the changes in carbon emissions. This analysis fills a significant gap in the literature by providing insights into the specific driving factors that influence carbon emissions in Central Asian countries. Our findings contribute valuable knowledge regarding the underlying determinants of carbon emissions in the region.

The ensuing segments of this paper are organized in the subsequent fashion. The second section introduces the research methodologies and sources of data utilized in the study. The third section exhibits results and discussions. The fourth section presents policy implications. And the fifth section shows conclusions.

## Methodology and data

### Emission accounts

The accounted emissions in this paper refer to territorial emissions, which are generated mainly from fossil fuel combustion. In this study, we adopt the IPCC administrative territorial approach to account for CO2 emissions in each country, which is recommended by IPCC (2006):

1 $$CE=\sum\nolimits_i\sum\nolimits_j{CE}_{ij}=\sum\nolimits_i\sum\nolimits_j{AD}_{ij}\times {NCV}_i\times {CC}_i\times {O}_i$$

where *CE* is the total amount of carbon emissions, which is the sum of carbon emissions from the fossil fuel type *i* in sector *j*; *AD*_*ij*_ measures the consumption of the fossil fuel type *i* induced by social and economic activities in sector *j*, which is also called activity data; *NCV*_*i*_ denotes the value of the net calorific of fossil fuel *i*, measuring the level of heat released in the burning process of this fossil energy; *CC*_*i*_ refers to the carbon content of fossil fuel *i*, showing the level of carbon emissions produced by per unit of heat burned from this fossil energy; and *O*_*i*_ denotes the oxidation effect of fossil fuel *i*, which is the proportion of carbon oxidized to CO_2_ during combustion.

### Tapio decoupling approach

Decoupling is used to describe whether the interdependence between variables lasts (Han et al. 2020). The low-carbon development mode is to achieve a negative growth of carbon emissions while maintaining positive economic growth, meaning that the nexus between carbon emissions and economic growth keeps thinning until it eventually vanishes (Chen and Yan 2022; Liang et al. 2022; Liu et al. 2022). In other words, economic development and carbon emissions are decoupled. Tapio decoupling index and OECD decoupling index are two widely used methods for decoupling in the current literature (Song et al. 2023; Wang et al. 2021a; Yang et al. 2023). The former directly measures the change rate of environmental quality as economic development changes, which introduces the concept of elasticity into decoupling indicators, while the latter adopts a more complicated relationship that can describe other marginal contributions (Wang et al. 2021b). In this study, we adopt the Tapio decoupling index method to measure the decoupling status of CO2-GDP in Central Asian countries. The decoupling coefficient *ε* can be measured as follows:

2 $$\varepsilon =\frac{\left({CO}_2^T-{CO}_2^0\right)/{CO}_2^0}{\left({GDP}^T-{GDP}^0\right)/{GDP}^0}=\frac{\Delta {CO}_2/{CO}_2^0}{\Delta \textrm{GDP}/{GDP}^0}$$

where ${CO}_2^T$ and *GDP*^*T*^ are the amounts of carbon emissions and GDP in the end year, and ${CO}_2^0$ and *GDP*^0^ are those in the first year. The decoupling coefficient *ε* denotes the change ratio of carbon emissions and GDP over a period of time. To be more specific, strong decoupling appears when carbon emissions continue to decrease when GDP keeps increasing, which is the most ideal state (Li et al. 2023a; Li et al. 2023b), while weak decoupling shows the opposite situation and means that carbon emissions grow positively under the condition of continuous economic recession.

### Index decomposition analysis

Index decomposition analysis (IDA) and structural decomposition analysis (SDA) are two methods commonly used in existing research for studying the decomposition between energy and the environment (Zhang et al. 2022). Compared with IDA, SDA has higher data requirements in that it is based on the input-output (IO) table data (Liu et al. 2021a). In addition, the logarithmic mean Divisia index (LMDI) in the IDA model has no residual and can effectively avoid the problem of pseudo-regression, which is a relatively complete decomposition method. Moreover, the LMDI-IDA method can effectively analyze the overall indicators and at the same time maintain the high consistency between various decomposition indicators (Ang and Wang 2015; Choi and Ang 2012; Ou et al. 2019; Shan et al. 2021). Therefore, it is one of the most widely used methods in carbon emissions decomposition (Alajmi 2021; Ang 2005, 2015; Hasan and Chongbo 2020; Hasan and Liu 2022; He et al. 2022). Combined with the Kaya identity, we decompose the driving factors of carbon emissions into the following five components: carbon intensity, energy consumption structure, energy intensity, economy, and population. The specific decomposition equation is shown as follows:

3 $${CO}_2=\sum\nolimits_j\frac{C}{E_j}\times \frac{E_j}{E}\times \frac{E}{GDP}\times \frac{GDP}{Pop}\times Pop= CI\times ES\times EI\times ECO\times POP$$

where *C* is the carbon emissions, *E* is the energy consumption, and so *E*_*j*_ is the energy consumed in sector *j*. Thus, *CI* and *ES* represent carbon intensity and energy consumption mix in sector *j*, respectively. *EI*, *ECO*, and *POP* refer to the energy intensity, economic growth, and population.

Furthermore, using the addition decomposition, we transform the above equation into the following one:

4 $$\Delta {CO}_2={CO}_2^T-{CO}_2^0=\Delta CI+\Delta ES+\Delta EI+\Delta ECO+\Delta POP$$

where ∆*CO*_2_ is the amount of carbon emissions changed from the first year to the end year, and ∆*CI*, ∆*ES*, ∆*EI*, ∆*ECO*, and ∆*POP* are the growths of corresponding factors during the same period which contributes to the carbon emissions change.

### Data

To calculate the carbon emissions generated during the process of economic activities, data on detailed energy consumption is necessary. On the one hand, as the sector types of final energy consumption vary dramatically in each country, we uniformly divide the sector types of final energy consumption into 47 sectors (see Supplemental Information Table S1), the same way as Liu et al. (2023), which is divided according to the International Standard Industrial Classification (United Nations 2008). On the other hand, we clarify fuel types into five categories: coal, oil products, natural gas, biofuels and waste, and crude oil (see Supplemental Information Table S1). All these energy consumption data are obtained from the Energy Balance Table in the National Statistical Committee of each Central Asian country.

To sum up, we utilize the data of six Central Asian countries during 2010–2020 to first investigate their territorial carbon emissions generated from fossil fuel combustion, as well as carbon emissions in different industrial sectors and from different energy types. We also pay attention to their decoupling states and the driving factors of carbon emissions.

## Results and discussion

### Overall carbon emissions trends

Overall, the total amount of carbon emissions in these six Central Asian countries increased from about 407 million tons (Mt) in 2010 to 548 Mt in 2020, with an annual average growth rate of 3.02%. In 2010, the proportion of carbon emissions in our sample countries to the world was 1.34%, which then increased to 1.74% in 2020 (IEA 2020), showing that the overall carbon emissions of Central Asian countries have a significant upward trend.

**Fig. 1:**
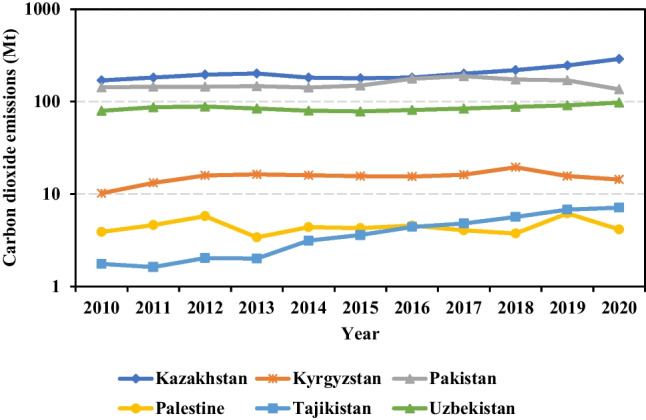
Carbon emissions of six countries in Central Asia during the period of 2010–2020

Figure 1 illustrates the emission trends (2010–2020) in six Central Asian countries, highlighting significant variations in carbon emissions. Kazakhstan and Pakistan exceed 100 Mt in total emissions, while Palestine and Tajikistan emit less than 10 Mt. In 2020, Kazakhstan, the largest emitter, produced 50 times more emissions than Palestine, the lowest emitter. Annual growth rates of carbon emissions differ greatly, ranging from 16.18% (Tajikistan) to − 0.04% (Pakistan).

In 2010, Kazakhstan accounted for 41.58% (169.29 Mt) of the total emissions in the region, increasing to 52.78% (289.30 Mt) in 2020. This significant contribution can be attributed to Kazakhstan’s large geographical area, population, economies of scale, and industrial output. To reduce emissions, the Kazakh government proposed a “green economy” strategy, aiming for a 40% reduction in emissions by 2050 compared to 2012 levels (Wang et al. 2019). Although the “green economy” plan reflects that the government attaches importance to low-carbon development, the energy transition process still takes some time.

Pakistan, ranking second among Central Asian countries, experiences high energy consumption in power generation, primarily from fossil fuel–based plants (Yousuf et al. 2014). Since 2017, carbon emissions in Pakistan show a slight download trend, which is linked with increased government promotion and acceleration of the carbon emissions mitigation process. The Pakistani government has continuously advocated that the electricity mix should transition toward nuclear and hydropower. Also, as Pakistan benefits from climate financing instruments such as clean development mechanism (CDM) and receives capital support from developed countries, it is gradually equipped with a higher ability to leapfrog to cleaner technologies and possesses more knowledge on carbon emissions mitigation (Bano et al. 2018; Mirza and Kanwal 2017; Yousuf et al. 2014). According to Mirza and Kanwal (2017), governments in Pakistan launched an environmental policy in 2005 to protect, conserve, and restore Pakistan’s environment and achieve sustainable economic development.

The reason for Uzbekistan’s relatively high carbon emissions is that, on the one hand, Uzbekistan’s power generation mainly depends on natural gas (Kochnakyan et al. 2013); on the other hand, the electricity generation infrastructures in Uzbekistan are outdated and obsolescent. Aminov et al. (2016) and Gómez et al. (2015) point out that 72% of thermal and hydroelectric power plants are built about 40 years ago, and 20% of the existing power plants have already exceeded their useful service life, resulting in traditional power plants’ energy utilization efficiency being just 30% (Gómez et al. 2015). These situations directly lead to high carbon intensity and a great deal of carbon emissions. In addition, as Uzbekistan is the most populated country in Central Asia (Ahunov et al. 2022; Bahrami et al. 2019; Djumaboev et al. 2019); the demand for a large amount of energy in people’s daily life may also be the potential reason for the high carbon emissions in Uzbekistan.

Carbon emissions in Palestine are small but fluctuate greatly, and the total amount of carbon emissions in 2020 was the lowest among all Central Asian countries, which is closely related to the unstable political and continuous war. Some communities in Palestine lack full civil and security control (Juaidi et al. 2016), and almost all petroleum products are imported through Israeli companies, which means that Israel takes full control of the energy import of Palestine. Furthermore, Palestine depends on other countries for 87% of its electricity imports. The high electric dependency also hinders the development of all energy and economic sectors. On the other hand, Palestine has abundant renewable energy resources, and some scholars advocate the development of renewables in Palestine (Abdallah et al. 2022; Hamed and Peric 2020; Juaidi et al. 2022; Kitaneh et al. 2012; Salah et al. 2021), such as strengthening the construction of wind turbines and photovoltaic power generation. For example, Abdallah et al. (2022) believed that wind power generation capacity in Palestine is rising. Moreover, Palestine has a high solar irradiation level and more than 3000 h of sunshine per year (Juaidi et al. 2016; Salah et al. 2021). Therefore, under the condition of shortage and strong instability of traditional energy, such as oil and gas, Palestine turns to developing clean and renewable energy, which is one of the reasons for its low carbon emissions and the emissions downward trend, and simultaneously it can also enable Palestine to enhance its energy security and independence ability.

Kyrgyzstan ranks at a moderate level for carbon emissions among the six Central Asian countries. Its abundant water resources, obtained from the vast Tianshan Mountain covering 90% of its territory, enable Kyrgyzstan to rely heavily on hydropower generation, thus reducing carbon emissions from thermal power plants (Mehta et al. 2022). Similarly, Tajikistan also benefits from abundant hydropower resources, contributing to its low carbon emissions. With the richest water resources in Central Asia, Tajikistan possesses advanced technological capacity and favorable economic conditions for water utilization, surpassing other Central Asian countries in this regard (Doukas et al. 2012; Karimov et al. 2013).

The above two sub-figures denote the carbon emissions intensity in 2010 and 2020, respectively; and the below two sub-figures denote the carbon emissions per capita in 2010 and 2020, respectively.

**Fig. 2:**
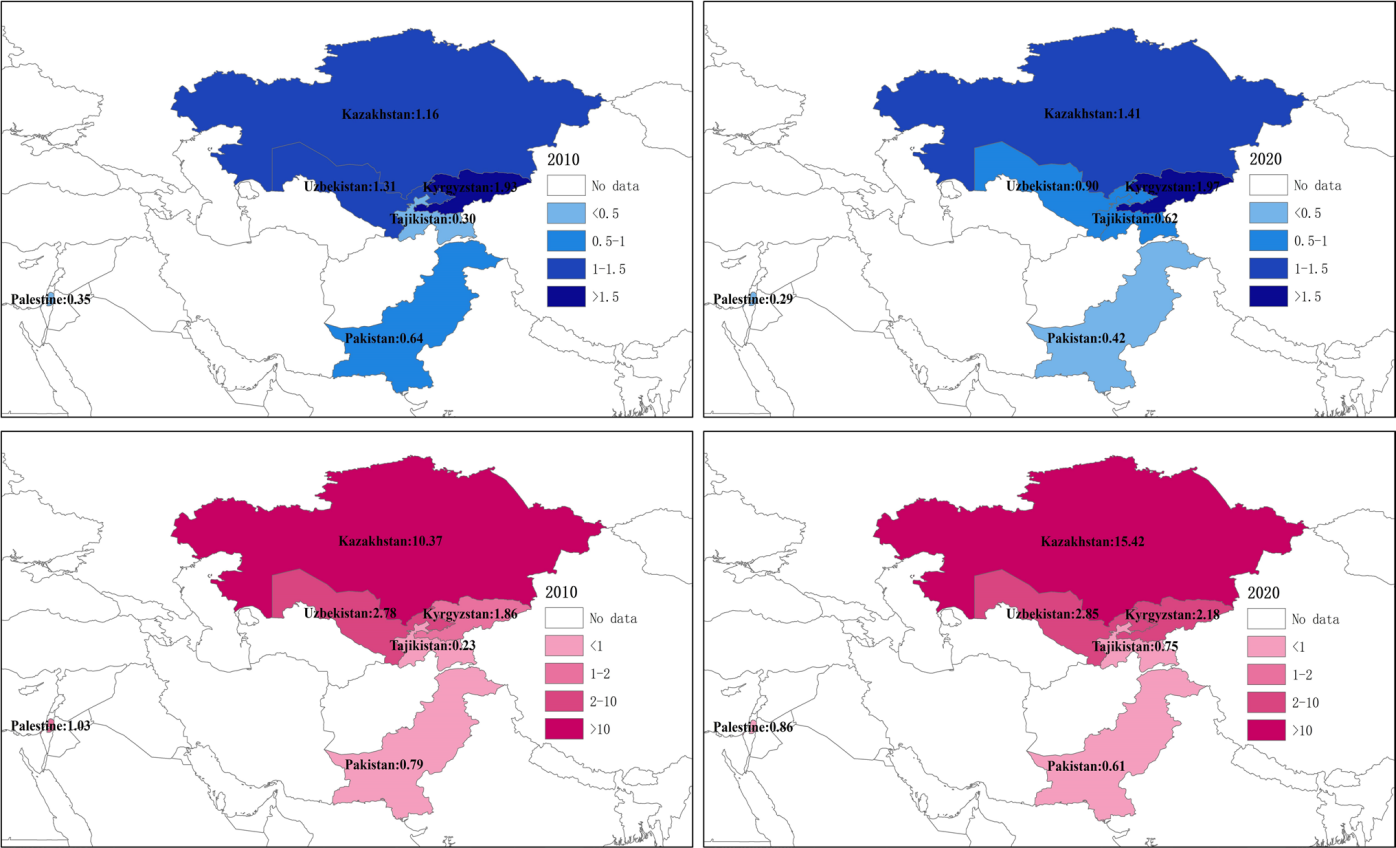
Carbon emission intensity and carbon emissions per capita in six Central countries

In addition to analyzing the overall carbon emissions, it is also necessary to combine carbon emissions with the economy (i.e., carbon intensity) and population (i.e., carbon emissions per capita). The above two sub-figures in Fig. 2 denote the carbon emissions intensity in 2010 and 2020, respectively; and the below two sub-figures in Fig. 2 denote the carbon emissions per capita in 2010 and 2020, respectively. The carbon intensity in Kyrgyzstan is the highest among the six Central Asian countries, despite the fact that its carbon emissions are at the middle level, implying that the negative environmental impact caused by the GDP per unit is large. In other words, the energy production efficiency and carbon efficiency are low, resulting in high pollution costs to support unit output value. Moreover, carbon intensity in Pakistan and Uzbekistan significantly decreased from 2010 to 2020. In terms of carbon emissions per capita, Kazakhstan’s per capita emissions far exceed those of other countries, which is also consistent with the findings from Fig. 1. By comparison, carbon emissions per capita in Pakistan are relatively low and decreased in the sample period.

**Fig. 3:**
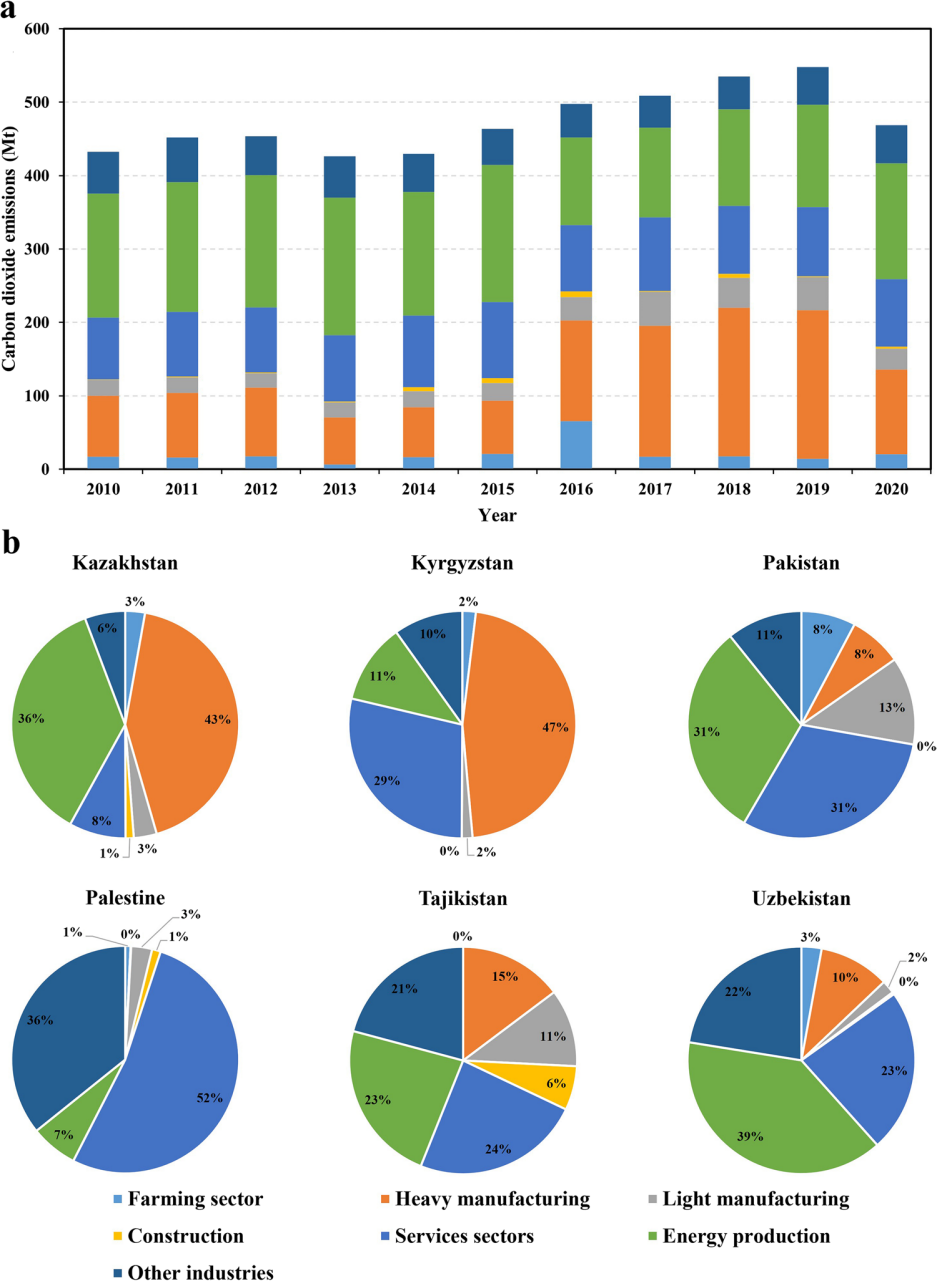
Sectoral emissions in all sample countries for the period 2010–2020 (**a**) and each sample country in all years (**b**)

Figure 3 presents the characteristics of average emissions in seven sectors by showing the proportion of each sectoral emission. Overall, heavy manufacturing, services sectors, and energy production are the three sectors producing top emissions. Heavy manufacturing accounts for more than 40% of carbon emissions in Kazakhstan and Kyrgyzstan. In Palestine, the carbon emissions of the service industry account for more than half of the carbon emissions of all industries, while in the other three central Asian countries, the carbon emissions of energy production account for a large proportion. The proportion of carbon emissions in energy production to all carbon emissions remains at approximately 35% in the study period. At the same time, the proportion of carbon emissions generated by the heavy manufacturing sector increased from 20 in 2010 to 40% in 2018, becoming the major carbon emission sector. During the period from 2016 to 2019, the carbon emission of the heavy manufacturing sector exceeded that of the energy production sector.

Among various sectoral emissions in Kyrgyzstan, the heavy manufacturing sector accounts for a large proportion, while the energy production sector accounts for a small proportion. The development of the heavy manufacturing industry in Kyrgyzstan mostly depends on traditional energy such as coal, oil, and natural gas. Kyrgyzstan is known as the “water tower” in Central Asia in that 90% of its territory is Tianshan Mountain. With sufficient water resources, Kyrgyzstan has developed mature hydropower-assisted electricity generation technology and can realize more than 90% of the country’s electricity supply through hydropower (Mehta et al. 2022).

Energy import and consumption are heavily and strongly affected by the political stability in Palestine, and nearly all products related to petroleum are dependent on and imported through Israel (Juaidi et al. 2016). Due to Palestine’s non-availability of natural resources, unstable political conditions, problematic border status, financial crisis, and weak industrial base, industry especially heavy industry in Palestine is very underdeveloped. Instead, service sectors especially the commerce and public sector are the primary consumers of energy and carbon emissions (Abdallah et al. 2022).

The sector that contributes most to carbon emissions in Pakistan is the energy production sector, including the production and supply of electric power, steam, gas, and hot water, which is also consistent with Yousuf et al. (2014) who found that the power sector in Pakistan was contributing more in greenhouse gas emissions. The electricity sector, one of the energy production sectors, is mainly generated by thermal power plants, which constitute 67.82% of the total electricity generation; although the electricity mix in Pakistan consists of thermal, hydro, and nuclear power plants, the last two kinds of power plants do not have primary status in the power generation systems. In view of the current situation of the power sector in Pakistan, it is necessary to use and deploy clean fuel and renewable energy so as to guarantee enough electricity demand while promoting the clean production of electricity. Yousuf et al. (2014) mentioned that wind, solar, and geothermal wave are potential and alternative renewable energy resources that deserve consideration in Pakistan.

Similar to Pakistan, Uzbekistan’s carbon emissions from energy production account for 39% of all carbon emissions, which is closely related to its reliance on traditional fossil energy, especially natural gas, for power generation. This finding is supported by Servert et al. (2014) who report that the energy mix in Uzbekistan is dominated by fossil fuel–fired thermal power stations, of which 85% is natural gas, 8% is heavy oil, and 7% is coal.

**Fig. 4:**
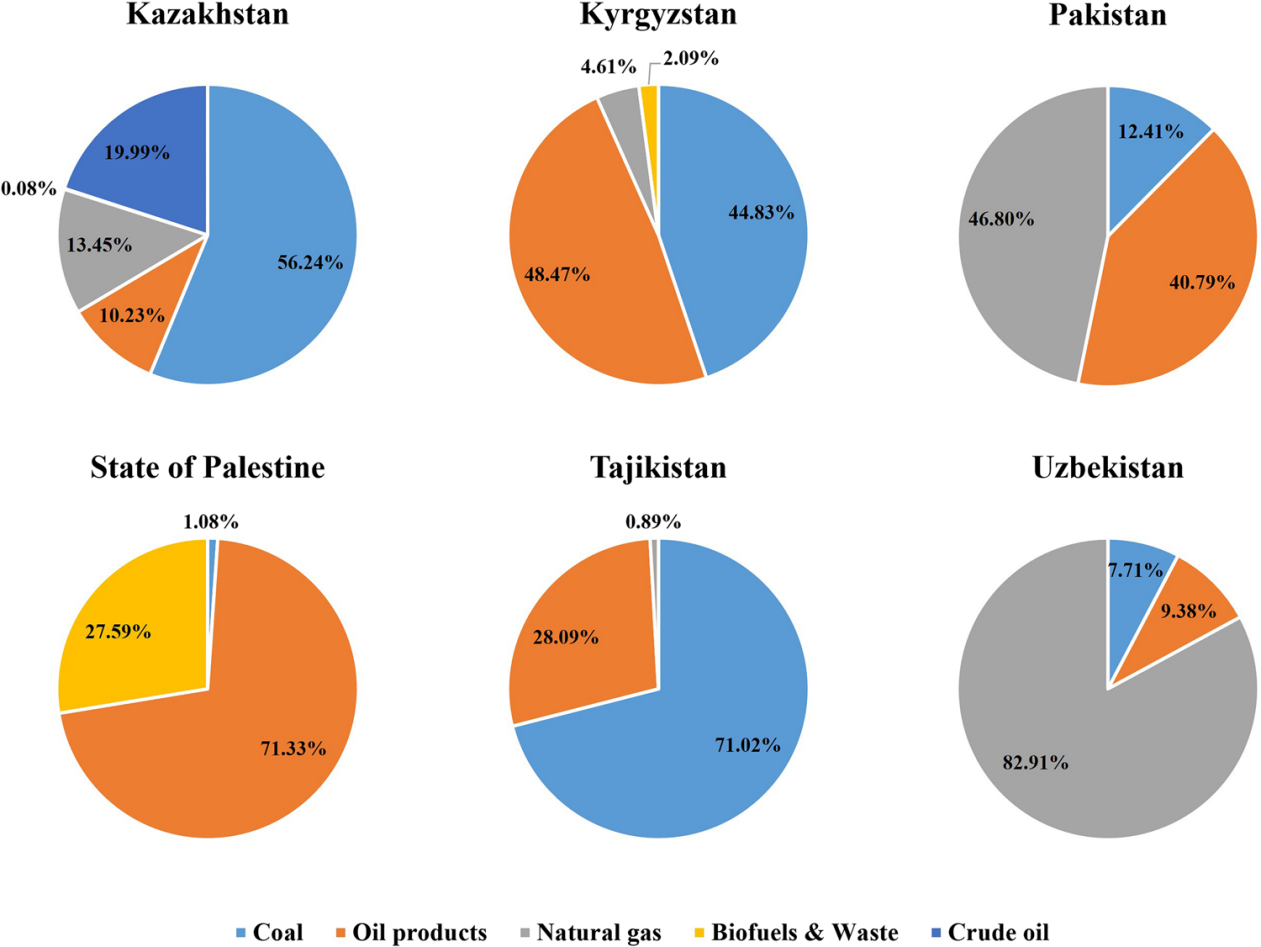
Energy structure of carbon emissions in each sample country during the period 2010–2020

As show in Fig. 4, coal is one of the most important sources of carbon emissions in Central Asian countries. The proportion of carbon emissions caused by coal ranges from 7.71 in Uzbekistan to 71.02% in Tajikistan. Notably, in Kazakhstan and Tajikistan, coal consumption accounts for more than half of the carbon emissions. Conversely, carbon emissions from the use of natural gas in Uzbekistan account for up to 82.91% of the total carbon emissions, indicating that Uzbekistan’s energy structure is dominated by natural gas.

The reason why coal-generated emissions are so large in Kazakhstan is that Kazakhstan is rich in energy resources, especially coal. This can also be seen and verified by Kazakhstan’s rich export of traditional energy, because Wang et al. (2019) point out that the annual export of coal, oil, and natural gas in Kazakhstan reaches 100 billion tons of oil equivalent. Unlike other Central Asian countries such as Pakistan and Palestine, Kazakhstan is a major energy exporter.

Similarly, coal is also the main energy source in Tajikistan, while oil and natural gas account for relatively low proportion. This is because the coal resources are richly reserved in Tajikistan, but it has few gas and oil resources (Karimov et al. 2013). On the other hand, other renewable energies except hydropower have not been fully utilized in Tajikistan (Doukas et al. 2012). In addition, Laldjebaev et al. (2018) reveal that the energy mix of Tajikistan gradually shifted and changed. Specifically, the proportion of natural gas once accounted for a larger share for a period of time, but has gradually decreased. In contrast, due to the increase in private vehicles and the increase of heat and power plants, the supply and demand of coal have made significant increases. And the explanation from Laldjebaev et al. (2018) is consistent with our findings.

Oil products are the main energy source of carbon emissions in Palestine, leading to a relatively single energy structure. Abdallah et al. (2022) also mentioned that a total of 81,903 TJ of energy was produced in 2019, of which around 85% were diesel, gasoline, kerosene, and LPG. The reasons for the low proportion of crude oil and natural gas in Palestine are two aspects: on the one hand, Palestine’s endowment of these two kinds of energy resources is not very large. On the other hand, unstable political background and a dependent energy sector (Juaidi et al. 2022) lead to high energy prices in Palestine, especially oil prices. Hence, the consumption of traditional energy such as crude oil in Palestine is scarce.

Coal and oil products are the main energy type in Kyrgyzstan. Yang et al. (2020) also point out that coal is the major fuel type in Kyrgyzstan while oil and gas are mainly imported. Moreover, Mehta et al. (2022) have explained the reasons why Kyrgyzstan does not fully use its potential renewable energy such as the government’s regulation on the minimal feed-in-tariff for all renewables and high focus on hydropower development. Our findings that the proportion of carbon emissions from the use of natural gas in Uzbekistan is 82.9% is consistent with the view of Bahrami et al. (2019) who show that natural gas accounts for approximately 82% of the total primary energy supply.

### Decoupling analysis

Table 1 shows the decoupling states between carbon emissions and GDP for six Central Asian countries. In general, the sample countries can be divided into four types according to their decoupling states.

**Table 1:** Decoupling nexus between carbon emissions and GDP based on the Tapio decoupling model

Region	Carbon dioxide emissions (Mt)		GDP (billion $,2015 constant price)		$$\frac{\Delta {CO}_2/{CO}_2^0}{\Delta \textrm{GDP}/{GDP}^0}$$		
	Range	Rate	Range	Rate	Sign	Value	Type
Kazakhstan	169.3–289.3	71%	146.6–205.8	40%	+/+	1.75	Expansive negative decoupling
Kyrgyzstan	10.2–14.3	41%	5.3–7.3	38%	+/+	1.08	Expansive coupling
Pakistan	142.6–135.6	− 5%	222.3–320.1	44%	−/+	− 0.11	Strong decoupling
Palestine	3.9–4.1	6%	11.1–14.0	27%	+/+	0.24	Weak decoupling
Tajikistan	1.8–5.6	306%	5.9–11.4	94%	+/+	3.27	Expansive negative decoupling
Uzbekistan	79.5–97.6	23%	60.9–108.2	78%	+/+	0.29	Weak decoupling

First, the growth rate of carbon emissions in Kazakhstan, Kyrgyzstan, and Tajikistan exceeds the economic growth rate, among which Kazakhstan and Tajikistan show expansive negative decoupling. This situation may be due to the fact that Kazakhstan and Tajikistan are both big coal consumers, and their energy structure transition is relatively backward. Therefore, it is important for these countries to recognize the dual development of economic transformation and the tendency toward low-carbon use. They should pay attention to the low-carbon management of investment, reduce their dependence on traditional fossil energy in the process of economic development, and improve energy utilization efficiency as well as the simple energy mix structure.

By comparison, Palestine and Uzbekistan show weak decoupling as their economic growth rates are in line with the increasing rates of carbon emissions. As mentioned above, Uzbekistan’s carbon emissions have maintained an upward trend in the past decade, while for Palestine energy security and economic development are in turmoil due to the unstable political situation (Hamed and Peric 2020; Juaidi et al. 2016). As such, they still need a lot of effort to achieve the decoupling of economic development and carbon emissions.

Pakistan has made great achievements in carbon decoupling on account that it has achieved positive economic growth under the condition of a negative emissions growth rate, becoming a strong decoupling country. The possible reasons may be that on the one hand, in recent years, Pakistan’s economic development has shifted from agriculture driven to industrial driven and has great momentum for development (Shahzad et al. 2017). On the other hand, although Pakistan consumes a large amount of traditional energy and generates carbon emissions in the electricity sector during the energy production process, policymakers and scholars are concerned about Pakistan’s renewable energy exploitation and exploration. Pakistan Energy Yearbook points out that governments are determined to enhance renewable energy capacity to about 20% of total electricity by 2025, while this figure in 2019 was only 16% (Raza and Lin 2022). Also, the government actively encourages the application of electric vehicles in different kinds of vehicles market such as light-duty vehicles, urban buses, and trucks (IEA 2020). Therefore, in Pakistan, we can see a promising decarbonization trend.

### Emission drivers

**Fig. 5:**
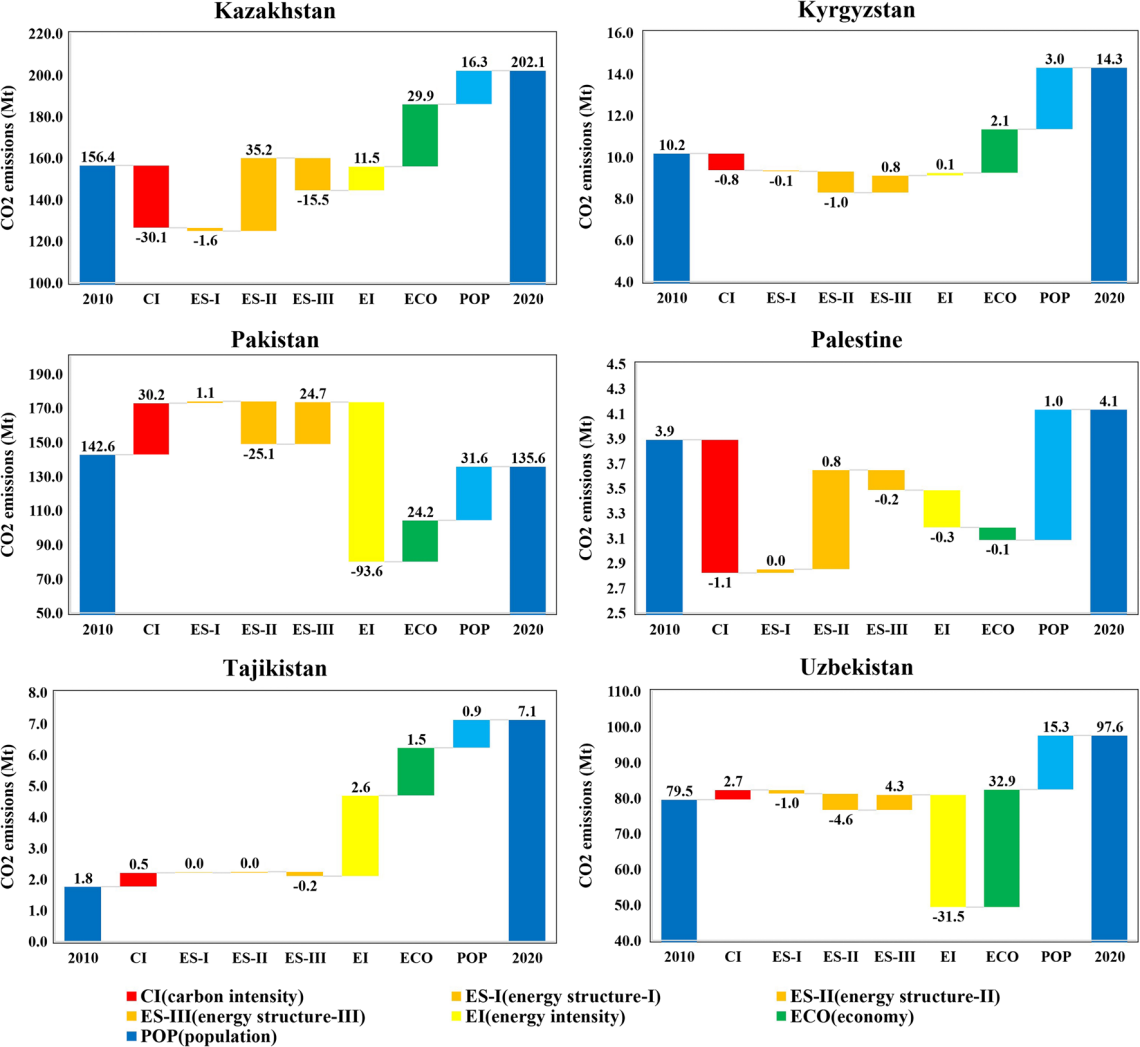
Drivers of emissions growth in each Central Asian country

We decompose the driving factors behind the carbon emissions changes and show the results in Fig. 5. Notably, the population is the major driver for the increased carbon emissions in all countries. The growth of carbon emissions in Central Asia caused by population has been confirmed by many scholars (Li et al. 2019; Lin and Raza 2021; Raza et al. 2023). Similarly, the economy also leads to more carbon emissions in these countries except for Palestine. In Palestine between 2010 and 2020, the economy causes a 0.1 Mt carbon emissions decrease, which is completely offset by the contribution of increasing carbon emissions from the population. In addition, the decrease in carbon emissions caused by Palestinian economic factors can also be attributed to its volatile political environment (Abdallah et al. 2022; Juaidi et al. 2016; Kitaneh et al. 2012).

The carbon intensity effect increased carbon emissions in Pakistan, Tajikistan, and Uzbekistan, while decreasing carbon emissions in the other three countries. Especially in Kazakhstan, the decreased carbon emissions caused by carbon intensity was 30.1 Mt, significantly and sufficiently offsetting the growth drivers of the economy. A similar situation can be found in Palestine in 2010–2015, where carbon intensity contributed to 1.1 Mt carbon emissions reduction, larger than the increased carbon emissions caused by the population drivers. Kazakhstan and Palestine, as typical expansive negative decoupling and weak decoupling countries, show an overall upward trend of carbon emissions during the past decade. The reason for the decrease in carbon emissions due to carbon intensity in Kazakhstan may be that the economic development is not only relying on traditional energy but also starting to develop renewable energy. For example, the Asian Development Bank pointed out that Kazakhstan is also gradually using blue hydrogen shifting away from natural gas (Asian Development Bank 2022).

In terms of energy intensity, it also varies widely across countries. Energy intensity plays the role of a positive driver in Kazakhstan, Kyrgyzstan, Tajikistan, but is a negative driver in other countries. In Palestine, energy intensity is the main reason for its carbon emissions changes. Specifically, the contribution of energy intensity amounted to a 93.6 Mt carbon emissions decrease. While in Tajikistan, energy intensity was responsible for the rise of its carbon emissions between 2010 and 2020. Moreover, although the energy intensity factor contributed 31.5 Mt to Uzbekistan’s carbon emissions reduction, it still did not reverse the overall upward trend of its carbon emissions. As shown in Fig. 1, carbon emissions in Tajikistan showed an upward trend during the study period, which is also accompanied by energy consumption. Although its economy was also growing, the growth rate of the economy was significantly lower than that of carbon emissions and energy consumption, and the economic growth rate was less than one-third of the carbon emissions growth rate (see decoupling results in Table 2). That is why Tajikistan’s energy intensity promoted the rise of carbon emissions.

We further decompose the energy consumption structure into three parts according to three sectors (i.e., agriculture, manufacturing industry, and services). It can be seen that among the three industrial sectors, the contribution of the primary sector, namely, agriculture, is very small in the change of carbon emissions, while the change in carbon emissions in the manufacturing industry is more remarkable and prominent, especially in Kazakhstan and Palestine. In these two countries, the energy consumption of the manufacturing industry led to an increase in carbon emissions. Moreover, the reduction of carbon emissions in Pakistan during the study period was not only caused by energy intensity factors as mentioned before but also contributed by the manufacturing industry factor. According to the Economic and Commercial Department of the Embassy of the People’s Republic of China in Kazakhstan, the government in Kazakhstan has strongly supported the development of the manufacturing industry. In the past decade, the output value of its pharmaceutical industry, machinery manufacturing industry, building materials industry, and chemical industry has kept rising, and the investment in fixed assets in these fields has also increased (Ministry of Commerce 2021). The investment and development may be the reason why energy consumption in the manufacturing industry contributes to its increased carbon emissions. Combining the decomposition and decoupling results in Palestine, Pakistan may experience industrial transformation and upgrading. On the one hand, the industrial transformation from the secondary industry to the tertiary industry has made its economic development and carbon emissions decoupled. On the other hand, the carbon emissions caused by the secondary industry decreased, while the carbon emissions caused by the tertiary industry increased. The Pakistan-China Economic Corridor (CPEC) significantly benefited the economic development and infrastructure construction in Pakistan, which also bring about considerable financial and foreign investments to Pakistan (Irshad 2015). In addition, the government in Pakistan attaches great importance to the country’s digital transformation and has launched a “Digital Pakistan” plan, laying a solid foundation for the development of the digital economy. In 2019, the National Bank of Pakistan issued the National Payment System Strategy (NPSS) to promote the construction of a modern digital payment network (Jamil 2021; Manzoor et al. 2021; Xin et al. 2023).

### Comparisons with other estimates

**Fig. 6:**
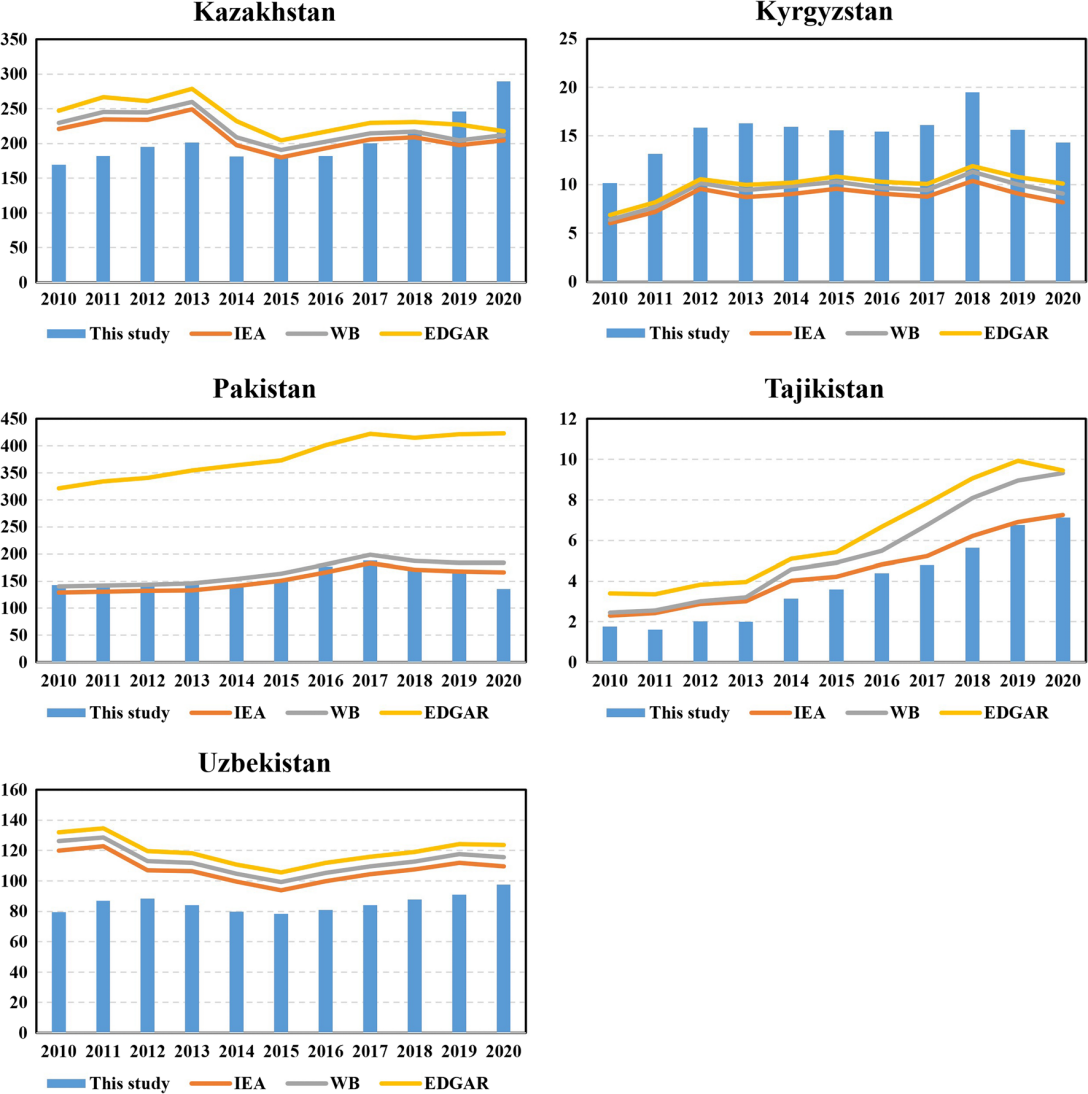
Carbon emissions of sample countries in World Bank database

To check the sensitivity and robustness of carbon emissions inventories compiled in this paper, we conduct a detailed comparative analysis between the calculated results in this study, and the emissions data in the World Bank database, IEA, and Emission Database for Global Atmospheric Research (EDGAR), which is shown in Fig. 6.

Notably, the statistics on carbon emissions in Palestine are not included in current databases. It is evident that the calculated carbon emissions presented in this paper generally tend to be lower than those reported in the current databases. This difference can be attributed to the following reason. The energy data we utilized is derived from the energy balance table published by the National Bureau of Statistics of each sample country. Thus, our calculations start from raw energy consumption and economic activity data. Moreover, we pay attention to specific and detailed emissions factors. For example, we consider several oil products (i.e., diesel, gasoline, kerosene, and LPG) and use corresponding emission factors, respectively. These emission factors are based on the default values provided by the IPCC. Consequently, our advantage lies in the utilization of more detailed original data and more granular emission factors, leading to more accurate estimates.

## Discussion

Based on our findings, we can put forward several targeted and practicable policy implications for future low-carbon and sustainable development in these Central Asian countries. First, to realize a low-carbon development model and achieve the decoupling of economy and carbon emissions, developing renewable energy to promote the energy structure transition is indispensable. That is to say, countries in Central Asia should actively increase the share of renewable energy sources in the country’s energy mix. To date, Central Asian countries have competitive advantages in renewable energy such as hydropower, hydrogen, wind energy, and solar energy; however, renewables have not been widely used and feasibly commercialized. Specifically, Tajikistan and Kyrgyzstan account for more than 80% of the freshwater resources in Central Asia, with huge hydropower potential. Palestine has 3000 sunshine hours per year, making solar energy a good development prospect in Palestine. Moreover, green hydrogen, as a typical decarbonization tool, can be abundantly and extensively exploited and utilized in Kazakhstan. And the Kazakhstan government also plans to reach 50% of the power generated by renewable energy of the total power output by 2050. In addition, the weak foundation of industry and renewable energy technology in Central Asian countries also restricts their large-scale deployment of renewable energy. Therefore, they also need to pay attention to technological breakthroughs and innovations in renewable energy. Moreover, countries whose carbon emissions are still high and have no obvious downward trend, such as Kazakhstan, as well as countries that have not been able to ratify intended NDCs, such as Kyrgyzstan and Uzbekistan, need to pay more attention to the difficulties encountered in energy transition, and actively change the original high-carbon industrial production and lifestyle. In contrast, Pakistan has entered the decoupling status, with a downward trend in carbon emissions, and has surpassed its mitigation contributions. Therefore, this country needs to keep developing its renewables and diversifying its energy mix.

Second, since these Central Asian countries may have some obstacles during the low-carbon production process, and lack experience in carbon emissions mitigation, it is important for developed countries to share more help and support during the process of energy transition and carbon emissions mitigation in these developing countries. Specifically, CDM and Nationally Appropriate Mitigation Actions (NAMAs), introduced in the Kyoto Protocol, advocate that developed countries finance clean energy projects in developing countries with the intention to meet their emission reduction commitments. For example, the CDM has given Pakistan great help. Hence, it is encouraged that more efforts and support need to be provided by developed countries with abundant resources and investments to Central Asian countries.

Third, while Central Asian countries plan to reduce carbon emissions, they also need to emphasize the issues of energy security and energy poverty. Put differently, they not only need to reduce their dependence on traditional energy but also need to reduce their dependence on imported energy by improving their renewable energy exploitation and exploration capacity, and ensuring that residents have easier access to reliable, clean, modern, and sustainable energy in their daily life. Palestine, in particular, needs to consider its severe issue of energy instability and dependence. Currently, electricity energy in Palestine is mainly purchased from other countries such as Israel, Jordan, and Egypt; only 4.1% of electricity is generated locally. Relying heavily on energy imports from other countries has seriously threatened the energy security of Palestine, and the fluctuation of energy prices has also been largely affected by the energy prices in the international market, which is not conducive to the stable development of the domestic economy. Simultaneously, these factors also lead to continuous fluctuate in carbon emissions. More seriously, not all Palestinian people have access to electricity the whole day, which means that it is not guaranteed for its people to obtain sufficient energy and the phenomenon of energy poverty exists pervasively. In addition, Kyrgyzstan’s energy sector suffers from significant crises to meet the growing energy demand of the country. Due to the geographical characteristic of Kyrgyzstan’s high altitude, although they have plentiful hydropower during summer, freezing conditions in winter lead to dramatically decreased hydroelectric generation, resulting in a demand for electricity significantly greater than the supply. In such circumstances, Kyrgyz people, especially those living in rural areas, can directly rely on conventional sources and low-efficient energy to cope with the harsh and extended winters.

## Conclusions

This study investigates the situation of carbon emission inventories in six Central Asian countries during the period of 2010–2020 in terms of 47 sectors and 5 types of fuels. The primary results highlight that the total carbon emissions of these countries vary greatly. Kazakhstan is the biggest emissions emitter with the highest level of carbon emissions per capita and the second highest level of carbon intensity. By comparison, Pakistan shows a brilliant carbon emissions mitigation trend with a − 0.04% average annual growth rate of carbon emissions. Heavy manufacturing and energy production contribute the most to carbon emissions, with the former demonstrating an upward trend. The energy mix is dominated by oil and natural gas.

The Tapio decoupling approach enables us to identify the decoupling states of these countries. Among them, only Pakistan exhibits a promising decarbonization trend. Palestine and Uzbekistan experience weak decoupling states as their economic growth rates exceed emissions growth rates. Kazakhstan, Kyrgyzstan, and Tajikistan face even worse conditions, as increased emissions growth rates outweigh increased economic growth rates.

The analysis of the driving factors shows that economic development and population growth contribute to emissions increases across all countries, while carbon intensity and energy intensity vary significantly. Energy intensity has a notable inhibiting effect on carbon emissions in Pakistan but leads to increased emissions in Kazakhstan and Tajikistan. Carbon intensity aids in significant emissions reductions in Kazakhstan and Palestine. Moreover, the analysis of energy consumption structure decomposition highlights the manufacturing sector as a significant driver of emissions growth in countries like Kazakhstan and Palestine.

There are several noteworthy research deficiencies in this study that require further attention and expansion. First, our study primarily concentrates on five energy types for carbon emissions accounting, but there is room for additional subdivisions within these categories to enable a more comprehensive measurement of carbon emissions resulting from the consumption of different energy sources. By delving into these subdivisions, we can gain a more detailed understanding of the specific contributions of each energy type to carbon emissions. Second, while we have successfully decomposed the factors that drive carbon emissions growth, our focus has been primarily on carbon intensity, energy consumption mix, energy intensity, economic growth, and population. However, it is crucial to acknowledge that there may be other significant factors that contribute to carbon emissions. Therefore, further research can further explore more drivers of carbon emissions growth.

### Supplementary material

ESM 1 Table S1. Economic sectors involved in six Central Asian countries’ energy statistics and CO_2_ emission inventory. Table S2. Energy types of six Central Asian Countries. (XLSX 13 kb)

## Data Availability

The data can also be downloaded freely from Carbon Emission Accounts and Datasets for emerging countries (CEADs) at www.cead.net.
